# Association of a Dietary Score with Incident Type 2 Diabetes: The Dietary-Based Diabetes-Risk Score (DDS)

**DOI:** 10.1371/journal.pone.0141760

**Published:** 2015-11-06

**Authors:** Ligia J. Dominguez, Maira Bes-Rastrollo, Francisco Javier Basterra-Gortari, Alfredo Gea, Mario Barbagallo, Miguel A. Martínez-González

**Affiliations:** 1 Geriatric Unit, Department of Internal Medicine and Geriatrics, University of Palermo, Palermo, Italy; 2 Department of Preventive Medicine and Public Health, University of Navarra-IDISNA, Pamplona, Spain and CIBER Fisiopatologia de la Obesidad y Nutricion (CIBERobn), Instituto de Salud Carlos III, Madrid, Spain; 3 Department of Internal Medicine (Endocrinology), Hospital Reina Sofia, Osasunbidea-IDISNA, Tudela, Spain; University College London, UNITED KINGDOM

## Abstract

**Background:**

Strong evidence supports that dietary modifications may decrease incident type 2 diabetes mellitus (T2DM). Numerous diabetes risk models/scores have been developed, but most do not rely specifically on dietary variables or do not fully capture the overall dietary pattern. We prospectively assessed the association of a dietary-based diabetes-risk score (DDS), which integrates optimal food patterns, with the risk of developing T2DM in the SUN (“Seguimiento Universidad de Navarra”) longitudinal study.

**Methods:**

We assessed 17,292 participants initially free of diabetes, followed-up for a mean of 9.2 years. A validated 136-item FFQ was administered at baseline. Taking into account previous literature, the DDS positively weighted vegetables, fruit, whole cereals, nuts, coffee, low-fat dairy, fiber, PUFA, and alcohol in moderate amounts; while it negatively weighted red meat, processed meats and sugar-sweetened beverages. Energy-adjusted quintiles of each item (with exception of moderate alcohol consumption that received either 0 or 5 points) were used to build the DDS (maximum: 60 points). Incident T2DM was confirmed through additional detailed questionnaires and review of medical records of participants. We used Cox proportional hazards models adjusted for socio-demographic and anthropometric parameters, health-related habits, and clinical variables to estimate hazard ratios (HR) of T2DM.

**Results:**

We observed 143 T2DM confirmed cases during follow-up. Better baseline conformity with the DDS was associated with lower incidence of T2DM (multivariable-adjusted HR for intermediate (25–39 points) vs. low (11–24) category 0.43 [95% confidence interval (CI) 0.21, 0.89]; and for high (40–60) vs. low category 0.32 [95% CI: 0.14, 0.69]; p for linear trend: 0.019).

**Conclusions:**

The DDS, a simple score exclusively based on dietary components, showed a strong inverse association with incident T2DM. This score may be applicable in clinical practice to improve dietary habits of subjects at high risk of T2DM and also as an educational tool for laypeople to help them in self-assessing their future risk for developing diabetes.

## Introduction

Type 2 diabetes mellitus (T2DM) is a pandemia of this century. This common chronic disease has increased massively in recent years in parallel with the obesity epidemic, affecting 382 million people worldwide in 2013 [1]. If the same trend continues, the estimates foresee that by 2035 there will be 592 million persons with T2DM, particularly among young adults and citizens from low- and middle-income countries [1–3]. These numbers are worrisome because T2DM is associated with substantial increased risk of cardio- and cerebrovascular events and mortality [4], as well as with severe disability due to blindness [5], chronic renal failure [6], and lower limb amputations [7]. The resulting mortality and disability entail overwhelming human, financial, and social burden [1–3]. The cost of preventing and treating T2DM may be high, but the cost of neglecting it will be vastly higher.

There is strong evidence demonstrating that dietary modifications may decrease incident T2DM by 33% [8], 50% [9], and 58% [10] in people at high-risk from China, Finland, and the USA, respectively. Moreover, the effect of the implemented dietary interventions in these three studies persisted in the long term [11–13]. Two recent systematic reviews evaluated available evidence for effectiveness including 53 studies [14] and cost-effectiveness including 28 studies [15] of combined diet and physical activity promotion programs concluding that these programs are effective in reducing new-onset T2DM, increasing reversion to normoglycemia, and improving diabetes and cardiometabolic risk factors in persons at risk. Programs that achieved a mean weight loss at one year of only 2.5% resulted in 60% reduction in diabetes development at 6 years [15].

Hence, it is essential to detect persons at risk for diabetes to implement intensive preventive interventions the earliest possible. Numerous diabetes risk models and scores have been developed but most are rarely used. A systematic review evaluated in detail 94 such models, testing 6.88 million participants followed for up to 28 years. Heterogeneity of the studies included in the review precluded meta-analysis. These predictive scores do not rely specifically on dietary variables or only include few nutritional items. Some scores may involve biochemical analytical tests not regularly available; other scores searching simplicity and practicality lack of completeness. Most scores/models are not focused on diet, include only few and general food components and do not fully capture the overall dietary pattern [16]. This is in contrast to the fundamental role of dietary habits as key determinants of obesity and T2DM [17].

Prospective cohort studies have provided evidence on the contribution of several specific dietary factors in the development of T2DM [18–20]. However, the total effect of all these dietary factors together has not been jointly evaluated to build an *a priori* dietary-based diabetes risk score. Therefore, we conducted the present analyses aiming to evaluate a dietary-based diabetes-risk score (DDS), and its association with incident T2DM, using data from the Mediterranean cohort of the SUN −Seguimiento Universidad de Navarra−project.

## Methods

### Study design and population

The SUN project is a prospective, permanently open, dynamic cohort of university graduates started in 1999 with biennial collection of updated information. The design and methods of the SUN study have been previously described in detail and can be found elsewhere [21,22]. The Institutional Review Board of the University of Navarra approved the study protocol. The initial response to a mailed questionnaire was considered as informed consent to participate.

For the present analyses, we examined the last available database as of December 2014, corresponding to 22,175 participants. We included participants who had spent enough time in the study (>2 years and additional 9 months) as to be able to complete and return at least the 2-year follow-up questionnaire; otherwise, they were excluded (n = 3108). Participants were excluded from the analyses if they reported total energy intake out of pre-defined limits [23] (n = 2089), or had a previous diagnosis of diabetes (n = 404). Some of them had more than one exclusion criteria. The final analytic population included 17,292 participants. Those with missing values in smoking (n = 575) were treated as another category (current/former smoker/never smokers/missing). Overall retention was 93.1% (93.1% of participants recruited at least 2 years and 9 months ago returned ≥1 of the follow-up questionnaires).

### Dietary assessment

Dietary habits were assessed at baseline by a semi-quantitative 136-item FFQ previously described in detail [24]. The validity [24,25] and reproducibility [26] of this questionnaire have been repeatedly reported. Nutrient scores were computed as previously described in detail [24–26] using the latest available Spanish food composition tables [27,28]. **Table 1** shows the foods and nutrients included in the diabetes score.

**Table 1:** Scoring criteria for the Diabetes Dietary Score in the SUN cohort, 1999–2014.

Component	Included foods
***Protection***	
• Vegetables	Carrot, pumpkin, Swiss chard, cabbage, cauliflower, broccoli, lettuce, chicory, escarole, tomatoes, green beans, eggplant, zucchini, cucumber, peppers, asparagus, spinach, other fresh vegetables
• Fruit	Citrus, banana, apple, pear, strawberry, peach, cherry, fig, melon, watermelon, grapes, kiwi, canned fruit
• Total dietary fiber	g/day
• Whole cereals	whole-grain bread
• Nuts	Almonds, peanuts, hazelnuts, pistachios, pine nuts, walnuts
• Coffee	Cups (50 ml) of coffee consumed
• PUFA	g/day
• Low-fat dairy	Skim or low-fat milk
• Alcohol(moderate consumption)	10 g/day for men and 5 g/day for women
***Increased risk***	
• Red meat	Beef, pork, lamb, liver
• Processed meat	Cooked ham, Parma ham, mortadella, salami, foie gras, spicy pork sausage, bacon, cured meats, hamburger, hot dog
• SSB	All sugar sweetened beverages

PUFA: Polyunsaturated Fatty Acids; SSB: sugar sweetened beverages

To build the dietary-based diabetes-risk score (DDS), we considered the consumption (g/d) of nine nutritional exposures which have shown associations with a decreased incidence of T2DM (vegetables, fruit, fiber, whole cereals, nuts, coffee, PUFA, low-fat dairy, alcohol in moderate amounts), and three food groups which have shown associations with an increased incidence of T2DM (red meat, processed meat, and sugar-sweetened beverages [SSB]) [18–20]. We adjusted the consumption of each nutritional variable for total energy intake by using the residual method separately for men and women [29]. The energy-adjusted estimates (residuals) were ranked according to their sex-specific quintile values (assigning a value of 1 for the first quintile, 2 for the second quintile, and successively until the value of 5 was assigned to the fifth quintile). The quintile values for the food groups with increased risk of incident T2DM were reversed (assigning a value of 5 for the first quintile, 4 for the second quintile, and successively until the value of 1 was assigned to the fifth quintile). For alcohol, 5 points were assigned for moderate consumption (10–50 g/day for men and 5–25 g/day for women); otherwise, the participant received zero points. To obtain the DDS, quintile values of nutritional variables with expected protection and reverse quintile values of food groups with expected increased risk were summed; thus, the final scores could range from 11 (lowest adherence) to 60 (highest adherence) points. We classified adherence to the DDS in 3 categories: low (11–24), intermediate (25–39), and high (40–60). We chose to use these specific round cut-offs points instead of quantiles because the groups thus built are more meaningful *per se* and could be more easily used for future comparisons with similar studies. This is in line with current recommendations given in epidemiology about procedures to categorize continuous variables [30]. Additionally, we also show the results of alternative analyses using absolute consumption cutoffs of the model (see below).

As sensitivity analyses, we built a similar model but considering the score as a continuous variable (for one and five additional points). We also repeated the analyses for men and women, separately; older (>50 years) and younger (<50 years) persons; higher (≥30 kg/m^2^) and lower (<30 kg/m^2^) BMI; changing the energy limits, including only participants with energy between percentile 1 and 99; and, excluding cases with an early diagnosis of T2DM during follow-up. We also built a similar score with the same exposures but using absolute cut-offs points for each one of the 12 food groups with goals expressed as servings/day or servings/week (i.e., normative or absolute cutoffs) [31,32] instead of using energy-adjusted categories of consumption. However, the same food groups as in the category-based assessment were considered and the score assigned 1 point for each of the 12 goals that was met (consumption of each item at or above the limit for protective foods and below the limit for deleterious foods). The cut-off points were as follows: vegetables (≥2/d), fruit (≥2/d), whole bread (>0/d), fiber (≥25 g/d), coffee (≥3/d), nuts (≥3/d), low-fat dairy products (≥1/d), PUFA (≥5 g/d), alcohol (>10 g <50 g/d for men; >5 g <25 g/d for women), meat (≤1/d), processed meat (≤1/d), SSB (≤1/d). In these analyses, we did not compute residuals from regressions on energy intake; instead, we adjusted for total energy intake by introducing it as a covariate in the standard multivariable models.

### Ascertainment of Diabetes

Detailed information on ascertainment of T2DM in the SUN cohort has been reported before [33]. In brief, we considered diabetes at baseline if participants reported a medical diagnosis or were receiving oral antidiabetic agents or insulin. We considered probable incident cases for participants who reported a T2DM diagnosis made by a doctor during follow-up. We sent additional specific questionnaires to these participants to confirm their diagnosis, and to specify further details (i.e., type, date of diagnosis, gestational diabetes, highest fasting glucose value, eventual OGTT, HbA1c, current use of oral antidiabetic agents or insulin, and occurrence of complications). Probable cases were requested to send us their medical reports detailing the diagnosis. A panel of physicians, blinded to dietary habits information, classified these medical records and adjudicated the cases as confirmed incident T2DM or not. As previously reported [33], the diagnosis criteria for confirmed T2DM cases were those of the American Diabetes Association.

### Other covariates

Covariates assessed at baseline included socio-demographic parameters (age, marital status, years of university education), anthropometric measurements (weight, BMI), health-related habits (smoking status, physical activity, sedentary lifestyle, hours sitting down, hours of television watching), and clinical variables (medications, personal history of hypertension, family history of diabetes). Self-reported weight and BMI have been previously validated in a sub-sample of this cohort [34]. Physical activity was assessed using a previously validated questionnaire with a Spearman correlation coefficient of 0.51 (p<0.001) with objective measurements [35]. Physical activity was expressed in metabolic equivalent tasks (METs-h/week) as calculated from the time spent at each activity in hours/week multiplied by its typical energy expenditure [36]. Adherence to the Mediterranean food pattern was appraised using the score proposed by Trichopoulou [37].

### Statistical analysis

For building the DDS, we used only the information from the baseline FFQ. Means with SDs for continuous baseline characteristics and proportions for categorical characteristics were calculated by categories of DDS. The time to the event was defined as the number of days from recruitment to the last questionnaire, death or a confirmed diabetes diagnosis as determined by the adjudicator, whichever came first. Cox proportional hazards analyses were fitted to assess the association of the DDS with incident T2DM. After a crude analysis, we fitted a model adjusted for sex and age (as the underlying time variable). In a subsequent model we additionally adjusted for major risk factors of T2DM (total energy intake, adoption of special diets, snacking between meals, baseline BMI, physical activity, hours of television watching, hours sitting down, smoking, marital status, personal history of hypertension, and family history of diabetes). Robust standard errors were used. All models were stratified by age groups and year of recruitment. The p for trend was calculated taking the median for each category and introducing this new variable as a continuous variable in the models. We evaluated the interaction between the dietary score and BMI through the likelihood ratio test for the fully-adjusted model with and without the product-term. Nested regression models after a stepwise forward selection algorithm were used to evaluate the contribution of each item to the final score. As the BMI is also related to dietary habits, we also evaluated the association between baseline BMI and the risk of T2DM in an ancillary analysis, using similar methods.

The analyses were performed with Stata software package version 12 (Stata Corp). All tests were two sided and statistical significance was set at P<0.05.

## Results

**Table 2** shows baseline characteristics of the studied population of participants in the SUN project, according to categories of DDS (low-to-high). Older participants, married participants, those with higher university years of education and more physically active participants were more likely to belong to the highest category of the DDS; whereas current smokers, and those with a higher total energy intake were more likely to belong to the lowest category of the DDS (**Table 2**). As expected, the consumption of the nine favorable nutritional factors (with the exception of PUFA intake) increased monotonically across increasing categories of the DDS, whereas the consumption of the three detrimental food groups monotonically decreased (P<0.001 for all) (**Table 2**). The most striking differences were observed for the consumption of whole bread, nuts (including all tree nuts and peanuts), low-fat dairy, fruits, and vegetables. The intakes of vitamin C, heme iron from heme sources, folate, and fiber were greater in the high score category group. Conversely, the intakes of total energy and total fat were lower in the high score category group. Although significant, only minimal differences were observed for carbohydrate and vitamin D intakes across DDS categories (**Table 2**). Analyses of the contribution of the different components of the DDS with nested regressions after a stepwise selection algorithm showed that the largest variability was explained by vegetables (18%) and low-fat dairy consumption (13%), with no single component apparently driving the score.

**Table 2:** Baseline categories of participants according to categories of the Diabetes Dietary Score (DDS) in the SUN (“Seguimiento Universidad de Navarra”) cohort, 1999–2014.

	Diabetes Dietary score (DDS)			
	Low (11–24)	Intermediate (25–39)	High (40–60)	p^1^
N	1180	12076	3893	
Sex, male (%)	40.3	39.6	43.1	0.001
Age (y)	32.1 ± 9.1^2^	37.8 ± 11.3	43.2 ± 12.4	<0.001
Marital status (married) (%)	38.7	50.9	58.6	<0.001
Years of university education	4.8 ± 1.3	5.1 ± 1.5	5.2 ± 1.6	<0.001
Family history of diabetes (%)	11.0	15.1	19.1	<0.001
Hypertension (%)	3.3	6.8	10.7	<0.001
High blood cholesterol (%)	8.2	16.1	24.1	<0.001
BMI (kg/m2)	22.7 ± 3.3	23.6 ± 3.5	23.9 ± 3.5	<0.001
Smoking (%)				
Current	25.3	22.5	20.0	<0.001
Former smoker	18.8	27.5	40.1	
Alcohol intake (g/d)	5.0 ± 13.3	6.2 ± 10.1	8.7 ± 9.4	<0.001
Physical activity (MET-h/wk)	18.6 ± 22.3	20.5 ± 21.6	25.8 ± 24.8	<0.001
Hours sitting down/d	5.9 ± 2.0	5.7 ± 1.9	5.5 ± 2.0	<0.001
TV watching (h/d)	4.5 ± 2.8	4.7 ± 2.7	4.7 ± 2.7	0.26
Vegetables (g/d)	320 ± 208	482 ± 297	720 ± 38	<0.001
Fruit (g/d)	183 ± 141	310 ± 271	497 ± 342	<0.001
Legumes (g/d)	22 ± 16	22 ± 18	24 ± 18	<0.001
Cereals (g/d)	122 ± 83	100 ± 73	100± 67	<0.001
Whole bread (g/d)	1.3 ± 9.1	9.4 ± 27	28 ± 42	<0.001
Potatoes (g/d)	27 ± 29	27 ± 29	29 ± 31	<0.01
Nuts (g/d)	3.4 ± 4.2	5.8 ± 8.9	13 ± 18	<0.001
Olive oil (g/d)	18 ± 15	18 ± 15	20 ± 15	<0.001
Meats/meat products (g/d)	250 ± 85	180 ± 73	132 ± 67	<0.001
Animal fats for cooking or as a spread (g/d)	1.7 ± 3.4	1.1 ± 2.7	0.7 ± 2.0	<0.001
Eggs (g/d)	28 ± 19	24 ± 16	20 ± 13	<0.001
Fish and other seafood (g/d)	84 ± 53	94 ± 58	114 ± 68	<0.001
Whole dairy products (g/d)	360 ± 251	204 ± 195	116 ± 138	<0.001
Low-fat dairy products (g/d)	97 ± 179	212 ± 240	308 ± 260	<0.001
Coffee (cups/d)	0.76 ± 1.1	1.16 ± 1.2	1.53 ± 1.3	<0.001
Following a special diet	2.7	6.9	14.0	<0.001
Between-meal snacking	44.0	33.6	26.2	<0.001
Dietary intakes				
Total energy (kcal/d)	2703 ± 544	2328 ± 616	2291 ± 610	<0.001
Carbohydrate (% of energy)	41 ± 7.2	43 ± 7.3	45 ± 7.6	<0.001
Protein (% of energy)	17.8 ± 3.2	18.2 ± 3.3	18.1 ± 3.3	0.002
Total fat (% of energy)	40 ± 6.0	37 ± 6.3	34 ± 6.6	<0.001
MUFAs (% of energy)	17 ± 3.3	16 ± 3.7	15 ± 3.8	<0.001
SFAs (% of energy)	15 ± 3.1	13 ± 3.0	10 ± 2.8	<0.001
PUFAs (% of energy)	5.1 ± 1.5	5.2 ± 1.5	5.1 ± 1.4	0.007
Vitamin C (mg/d)	187 ± 86	258 ± 138	374 ± 178	<0.001
Vitamin D (mcg/d)	3.5 ± 2.2	3.6 ± 2.4	4.2 ± 2.8	<0.001
Iron from heme sources (mg/d)	16 ± 4	16 ± 5	19 ± 6	<0.001
Folate (mcg/d)	307 ± 108	383 ± 158	521 ± 194	<0.001
Dietary fiber (g/d)	20 ± 7	26 ± 11	36 ± 14	<0.001

MET: metabolic equivalent task; MUFA: monounsaturated fatty acid; SFA: saturated fatty acid; PUFA: polyunsaturated fatty acid

^1^ Comparisons of characteristics across categories of the diabetes dietary score were performed by using 1-factor ANOVA for quantitative variables or chi-square tests for categorical variables.

^2^ Mean ± SD (all such values)

During 159,567 person-years follow-up (mean follow-up: 9.2 years; range: 1.6–15.3 years), we confirmed 143 first diagnoses of T2DM among 17,292 participants of the SUN project. A significantly inverse linear trend in the Cox model was apparent for the association between DDS and the risk of T2DM after adjustment for sex and age (P = 0.004) (**Table 3**). The fully-adjusted HRs (95% CIs) for categories of intermediate and high adherence compared with the low adherence category (reference) were 0.43 (0.21, 0.89), and 0.32 (0.14, 0.69), respectively, with a significant inverse linear trend (P = 0.019) (**Table 3**). The DDS and the Mediterranean diet score [37] had a Spearman correlation coefficient of 0.617 (p<0.001), suggesting a moderate degree of overlapping between both *a priori*-built dietary scores.

**Table 3:** HRs (95% CIs) of incident diabetes according to baseline categories of the Diabetes Dietary Score in the SUN cohort, 1999–2014^1^.

	Diabetes Dietary Score			
	Low (11–24)	Intermediate (25–39)	High (40–60)	p
N	1180	12076	3893	
n of incident diabetes	10	99	34	
Persons-year of follow-up	11793	113284	34490	
Age-adjusted diabetes incidence (x 10^−3^)	0.85 (0.41, 1.56)	0.45 (0.23, 0.86)	0.32 (0.16, 0.67)	
Crude HR*	1 (ref)	0.47 (0.24, 0.93)	0.32 (0.15, 0.66)	0.006
Age-, year of recruitment and sex-adjusted HR	1 (ref)	0.46 (0.23, 0.91)	0.30 (0.15, 0.64)	0.004
Multivariate-adjusted HR^1^	1 (ref)	0.43 (0.21, 0.89)	0.32 (0.14, 0.69)	0.019

* Age as the underlying time variable.

^1^ Adjusted for sex, total energy intake, following a special diet, snacking between meals, BMI, physical activity, hours of television watching, hours sitting down, smoking, marital status, personal history of hypertension, and family history of diabetes (parents and/or siblings).

**Fig 1:**
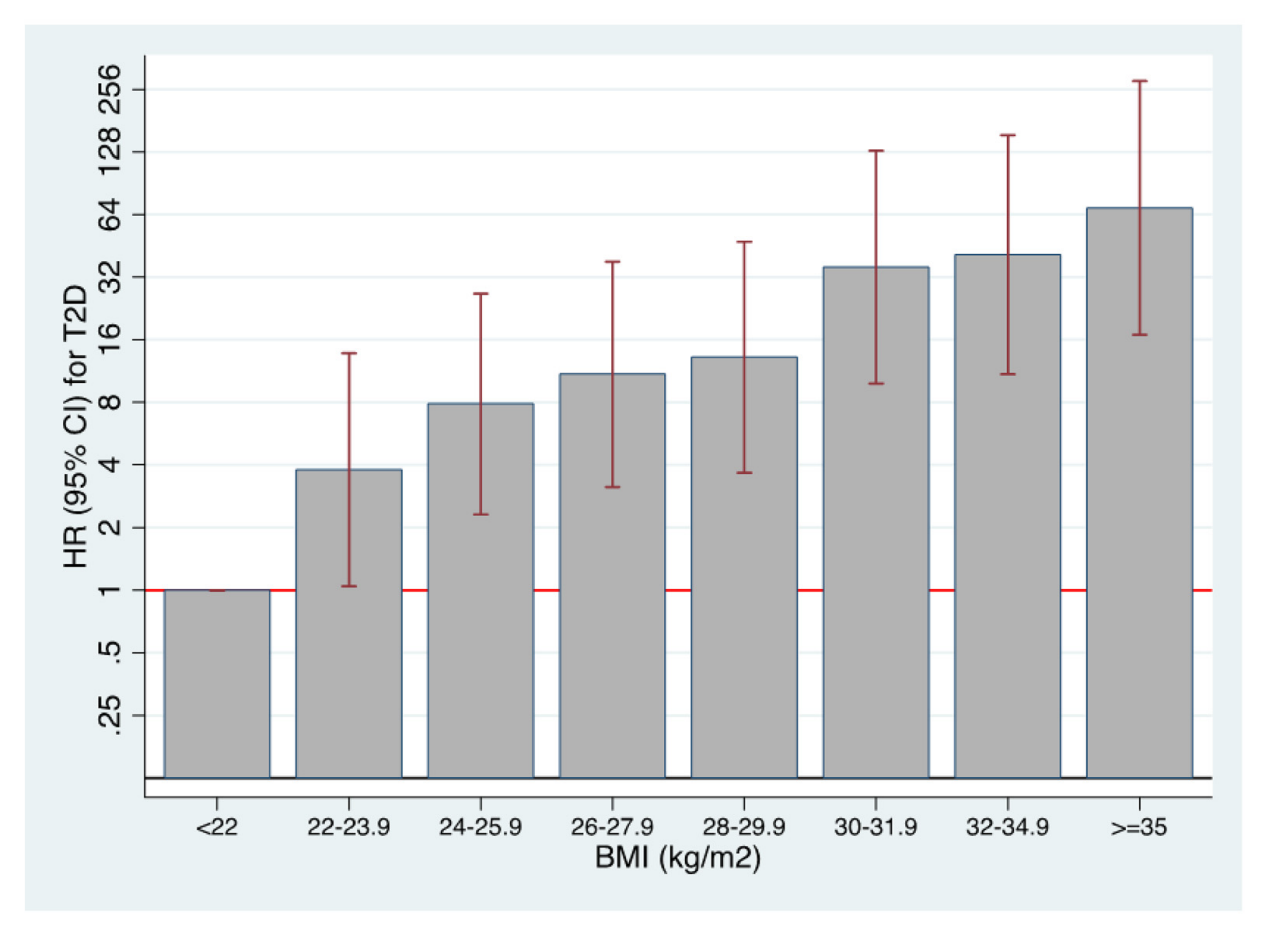
Risk of developing T2DM and BMI. Multivariable-adjusted hazard ratios for the risk of developing T2DM according to baseline body mass index. The SUN cohort 1999–2014.

In the ancillary analysis of the association between BMI and T2DM, we found that a BMI>35 comported a 64-fold increase in incident diabetes compared to a BMI<22 (**Fig 1**); this strong effect persisted when considering separately men (25 fold) and women (56 fold), with higher increases for women, and when considering participants younger (69.5 fold) and older than 50 years (59 fold). There was a continuous rise in the risk of diabetes as the BMI increased. Interestingly, the increased risk (4 fold) was already evident when comparing persons with BMI from 22.1 to 24.9 versus those with BMI<22 (**Fig 1**).

**Fig 2:**
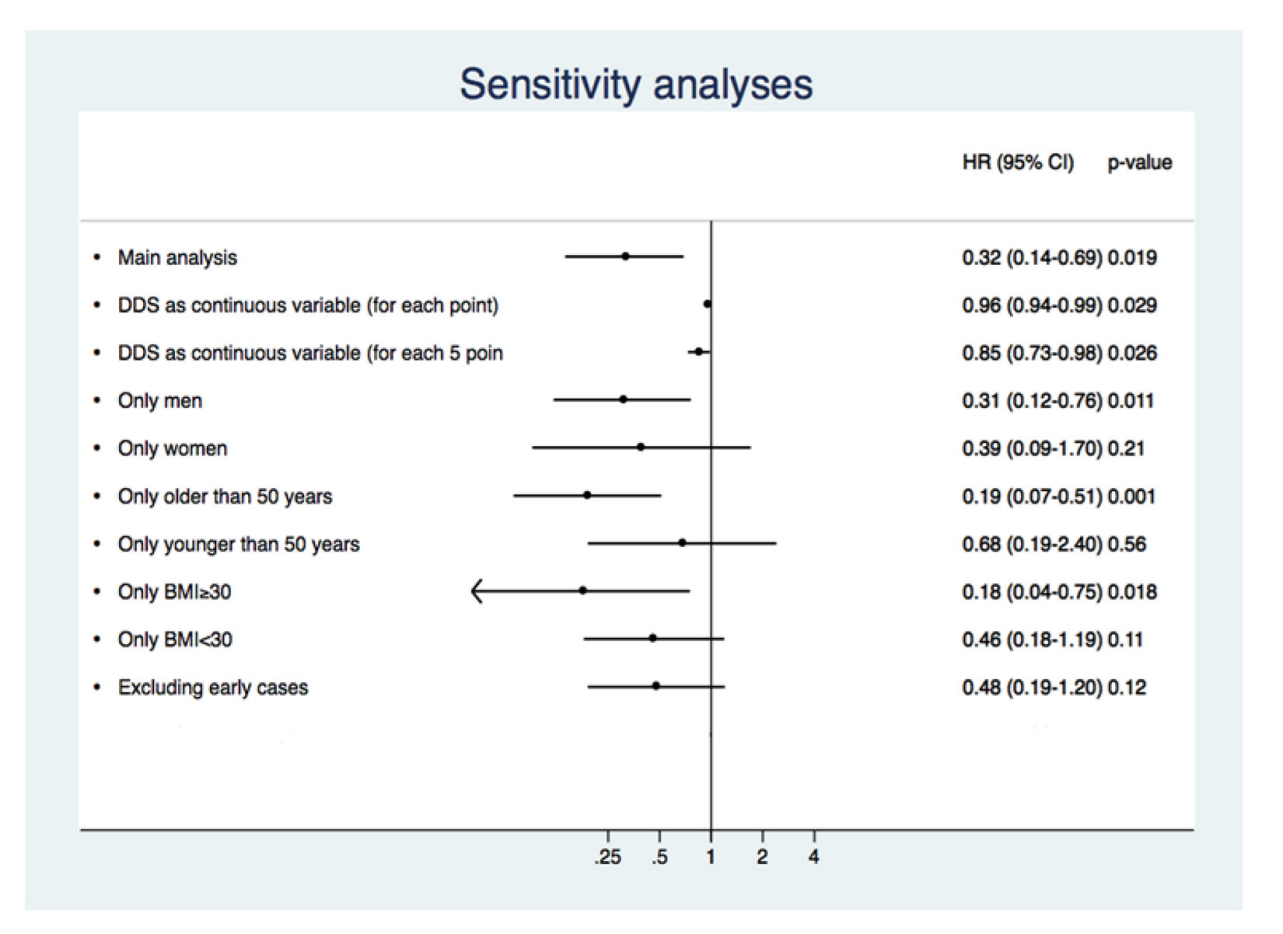
Risk of developing T2DM and DDS. Sensitivity analyses of the association between the dietary score and the risk of incident diabetes. Multivariable adjusted model. The SUN Project 1999–2014.

Several sensitivity analyses were carried out in order to appraise the strength of our findings (**Fig 2**). Assessing DDS as a continuous variable, for each five additional points, the risk of diabetes decreased by 15%. When we assessed men and women separately, for both groups the dietary score was inversely associated with T2DM risk in multiple-adjusted models; the comparison of high vs. low category was significant for men, but not for women. Considering participants older and younger than 50 years separately, for both groups the fully-adjusted DDS showed an inverse association; the comparison of the high vs. low category was significant for participants older than 50 years, but it was not significant for participants younger than 50 years. Examining separately participants with BMI higher and lower than 30, for those with BMI≥30 comparison of the high vs. low category was significant, while it was also inversely associated with T2DM for those with BMI<30 without reaching statistical significance. No significant interaction was observed between the score and BMI, when we dichotomized BMI by 30 kg/m^2^, or when we considered BMI as a continuous variable in the interaction product-term. After excluding early cases of T2DM (those diagnosed during the first 2-year follow-up period), the score was still inversely associated with T2DM, but without reaching statistical significance.

**Fig 3:**
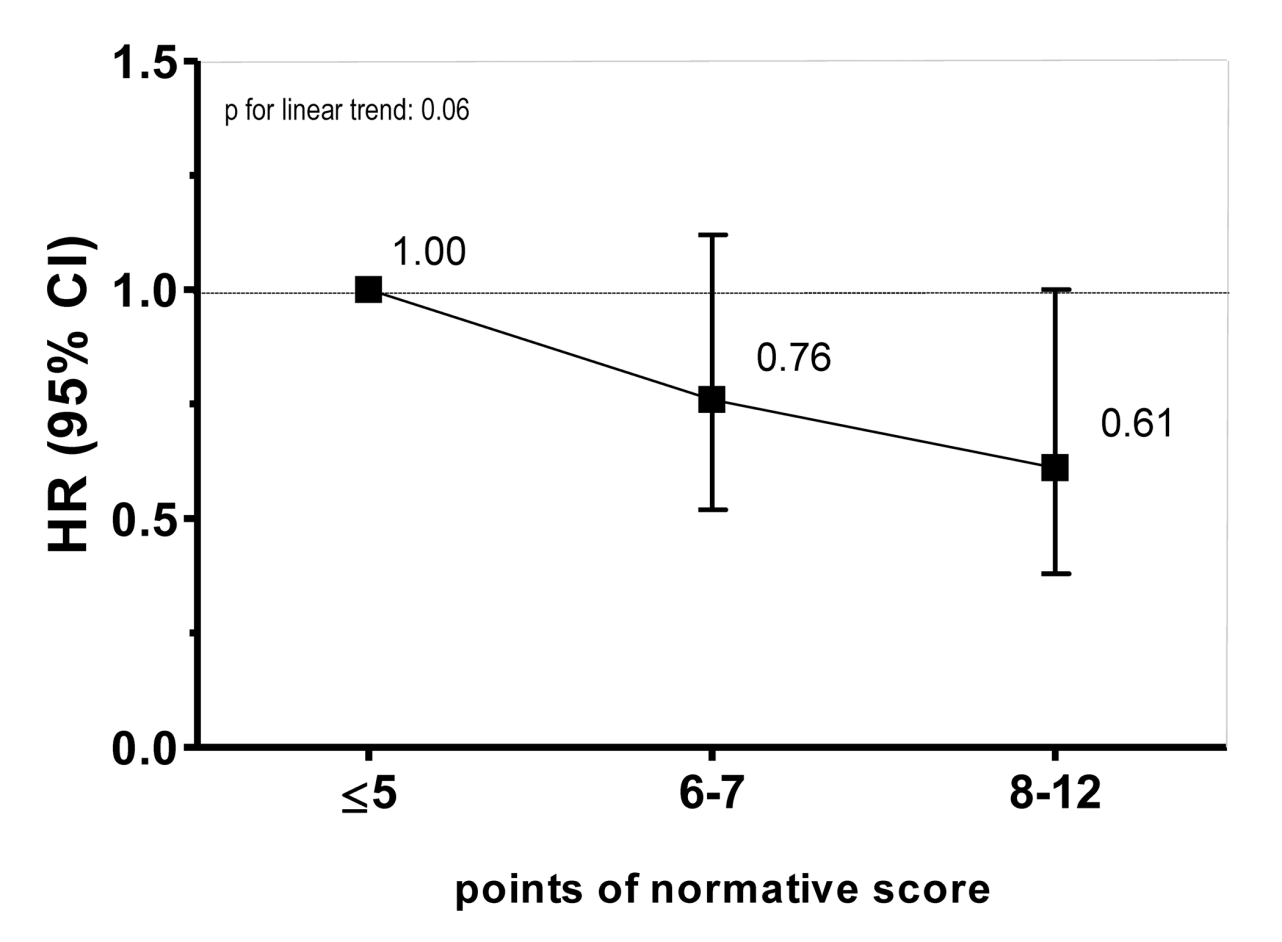
Hazard ratios (HR) and 95% confidence interval (CI) for incident T2DM according to a DDS. For this analysis we used servings/day or servings/week (i.e. normative or absolute cut-off points [31,32]), assigning one point to each of the 12 goals accomplished. Results represent a fully adjusted model, adjusted for age (as the underlying time variable), sex, total energy intake, following a special diet, snacking between meals, BMI, physical activity, hours of television watching, hours sitting down, smoking, marital status, personal history of hypertension, and family history of diabetes [parents and/or siblings]).

Finally, we constructed a similar diabetes dietary score using servings/day or servings/week (i.e., normative or absolute cut-off points [31,32] instead of using energy-adjusted categories of consumption). Because this score assigned one point to each of the 12 goals accomplished, its possible range was 0–12 points. The fully-adjusted HRs in this sensitivity analysis is shown in **Fig 3**.

## Discussion

We assessed an *a priori* dietary-based diabetes score (the DDS) to appraise the association of the total dietary pattern with type 2 diabetes. This score, in contrast with previous models, was based on several specific dietary components with available previous evidence of their association with increased or decreased risk of T2DM. This is important because of the key role of dietary habits as determinants of obesity and T2DM. It may represent a useful tool, not only for identification of high-risk individuals according to their dietary pattern, but also to educate consumers on healthy dietary and lifestyle choices while assessing their risk of diabetes.

To construct our DDS, we used dietary factors proved to contribute to T2DM risk [18–20]. Therefore, there was a rationale to incorporate these elements to build an *a priori* dietary model. These factors represent established risk parameters, but some of them have not been combined in a single score nor even included in previous diabetes risk assessment models [16]. While only modifiable risk factors can be addressed by interventions, non-modifiable risk factors, like age and family history of T2DM, contribute significantly to determine a person’s risk and were all included in our analyses as potential confounders.

We found a significant, but moderate correlation between the DDS and the Mediterranean diet score (MDS). Although the MDS was not built to combine nutritional factors reported to be associated with T2DM, it has been consistently associated with lower incidence of T2DM [31,33,38]. Some of the components are shared by the DDS and the MDS, but, there are some differences between both scores. For example, we did not include legumes in the DDS because there is still no report showing relevant association of legumes intake with incident diabetes. Instead of including cereals we included total fiber and whole grains because both have been associated with a reduced diabetes risk [18,39]. Dairy products are considered detrimental in the MDS, while there is evidence supporting an inverse association of some dairy products, especially low-fat dairy, with diabetes risk [18,19], which we included in the DDS. We also included PUFA and coffee intake because both were reported to be associated with lower diabetes risk [18,20]; SSB are not considered in the MDS, but there is evidence on their association with an increased risk of diabetes [18].

A number of diabetes risk scores are now available providing a fairly good but not perfect estimate of the probability to develop diabetes in the years ahead. In a review of 94 T2DM risk assessment models, only seven were suitable for clinical practice [16]. Most models claimed wide applicability, which is difficult to reconcile with the inherent selection bias of the sample/cohort characteristics used to develop many scores. It appears that there is a recent shift in priorities from the exclusive chase of statistical brilliance to the practical application and outcomes of using diabetes risk scores in real-world prevention programs [16,40]. We aimed to assess whether a score exclusively based on the potential role of nutritional elements may show a strong association with incident T2DM. Probably, there is no single ideal risk score universally applicable, as the value depends not only on its statistical properties but also on its context of use, which may define which data are available to consider. Perhaps development of national/regional models and risk categories, according to the local/regional characteristics and traditions is advisable. Extrapolation of scores in different contexts/populations showed variability in estimates of risk as high as twenty-fold [41,42]. The model we propose is inexpensive, can be self-administered, and it is directed to educate laypeople and to encourage persons at risk to adopt/improve their healthy dietary choices. These healthy choices, proved to prevent T2DM, may also influence the incidence of other non-communicable diseases [43], therefore, a widespread dissemination of these type of self-assessment is warranted.

Several RCTs [8–14,44] have shown that weight reduction should be the primary goal of diet and lifestyle interventions addressed to prevent T2DM. In the Nurses’ Health Study, T2DM risk over a 14-year follow-up was 49-fold higher among women with baseline BMI>35 vs. those with BMI<22 [45]. Participants of the US male health professionals cohort with BMI≥35 had RR of incident diabetes 42-fold higher than those with BMI<23, after adjustment for confounders [46]. We found an even higher relative risk of T2DM according to BMI in our younger SUN cohort, with a 64-fold increase in incident diabetes for BMI>35 vs BMI<22. However, aging is a substantial risk marker and the absolute risk will be always higher with age [47]. Importantly, the risk of diabetes was increased by ∼4-fold for persons with BMI from 22.1 to 24.9 vs. BMI<22, emphasizing the key role of achieving and maintaining an ideal BMI as early as possible and highlighting the perils of a slightly increased BMI within the *normal* range. We included this assessment together with the DDS in order to provide a perspective on the relative roles of dietary composition and BMI on the risk of developing T2DM. Although being too thin or losing weight rapidly is associated with higher mortality risk among older persons [48], a recent study showed no evidence of protection for overweight/obesity on mortality in older persons with T2DM who never smoked [49].

The strengths of our study are: a) large sample size; b) high retention rate; c) prospective design; d) lengthy follow-up; e) ability to control for a wide array of confounding factors, including potential lifestyle and demographic confounders; f) inclusion of several sensitivity analyses where the results still pointed to a negative association in their point estimates (though confidence intervals were wide because of the reduction in sample size caused by splitting the sample).

Potential limitations include: a) self-reported information, however, parameters such as self-reported weight and BMI and the FFQ have been previously validated in sub-samples of this cohort [34]; b) the cohort is composed of middle-aged, highly educated persons, with low prevalence of overweight/obesity and high level of physical activity. This explains the relatively low number of observed cases and the consequent width of some confidence intervals. However, we found strongly significant inverse associations for DDS and strongly significant direct associations for BMI; c) though SUN cohort is composed of highly educated participants, regardless of this fact, the generalizability of our results must be based on common biological mechanisms instead of on statistical representativeness; we used restriction to reduce potential confounding by disease, education, socioeconomic status, and presumed access to health care; however, future studies are needed in order to test the applicability of our results to other populations; d) finally, concerns may arise from the use of FFQ, which may be subject to information bias. However, the FFQ used has been repeatedly validated [24–26]; furthermore, it is difficult to find a better alternative method to characterize food habits of large samples of persons, followed over long periods of time, in order to assess associations with incident clinical end-points [23].

In conclusion, a score exclusively based on dietary components showed a strong inverse association with incident T2DM. This score may be applicable in clinical practice because it is based on variables that can be gathered in primary care, and this score may be even gathered using self-administered tools. Furthermore, it may well be an educational tool for laypeople to self-assess their risk of diabetes. Future studies are warranted in order to test whether the application of this model may be able to help modify dietary choices, incident T2DM and related morbidity.

## Supporting Information

S1 File STROBE checklist for cohort studies. (DOC)
