# Supplementary material for: Association of a Dietary Score with Incident Type 2 Diabetes: The Dietary-Based Diabetes-Risk Score (DDS)
Source: PLoS One. 2015 Nov 6;10(11):e0141760. doi: 10.1371/journal.pone.0141760 (PMC4636153; doi:10.1371/journal.pone.0141760)
Supplement: S1 File — (DOC) [file pone.0141760.s001.doc]

STROBE Statement—Checklist of items that should be included in reports of ***cohort studies***

|  | Item No | Recommendation |
| --- | --- | --- |
| **Title and abstract** | 1 | (*a*) Indicate the study’s design with a commonly used term in the title or the abstract  PAGE 2, Abstract, Methods |
|  |  | (*b*) Provide in the abstract an informative and balanced summary of what was done and what was found  PAGE 2 |
| Introduction | | |
| Background/rationale | 2 | Explain the scientific background and rationale for the investigation being reported  PAGES 4 |
| Objectives | 3 | State specific objectives, including any pre-specified hypotheses  PAGE 4, last paragraph |
| Methods | | |
| Study design | 4 | Present key elements of study design early in the paper PAGE 5, first paragraph |
| Setting | 5 | Describe the setting, locations, and relevant dates, including periods of recruitment, exposure, follow-up, and data collection PAGE 5, first paragraph |
| Participants | 6 | (*a*) Give the eligibility criteria, and the sources and methods of selection of participants. Describe methods of follow-up PAGES 5, first and second paragraph |
|  |  | (*b*) For matched studies, give matching criteria and number of exposed and unexposed NOT APPLICABLE |
| Variables | 7 | Clearly define all outcomes, exposures, predictors, potential confounders, and effect modifiers. PAGE 5, third paragraph, PAGE 6, PAGE 7, PAGE 8  Give diagnostic criteria, if applicable PAGES 7, last paragraph, PAGE 8, first paragraph |
| Data sources/ measurement | 8* | For each variable of interest, give sources of data and details of methods of assessment (measurement). PAGE 5, third paragraph, PAGE 6, PAGE 7, PAGE 8  Describe comparability of assessment methods if there is more than one group NOT APPLICABLE |
| Bias | 9 | Describe any efforts to address potential sources of bias PAGE 14, second paragraph, PAGE 16, first paragraph |
| Study size | 10 | Explain how the study size was arrived at PAGES 5, second paragraph |
| Quantitative variables | 11 | Explain how quantitative variables were handled in the analyses. If applicable, describe which groupings were chosen and why PAGES 6-7 |
| Statistical methods | 12 | (*a*) Describe all statistical methods, including those used to control for confounding PAGES 9 |
|  |  | (*b*) Describe any methods used to examine subgroups and interactions PAGES 9 |
|  |  | (*c*) Explain how missing data were addressed PAGE 5, second paragraph |
|  |  | (*d*) If applicable, explain how loss to follow-up was addressed PAGE 5, second paragraph |
|  |  | (*e*) Describe any sensitivity analyses PAGE 7, second paragraph |
| Results | | |
| Participants | 13* | (a) Report numbers of individuals at each stage of study—eg numbers potentially eligible, examined for eligibility, confirmed eligible, included in the study, completing follow-up, and analysed PAGE 5, TABLE 2 |
|  |  | (b) Give reasons for non-participation at each stage PAGES 5 |
|  |  | (c) Consider use of a flow diagram NOT APPLICABLE |
| Descriptive data | 14* | (a) Give characteristics of study participants (eg demographic, clinical, social) and information on exposures and potential confounders TABLE 2 and PAGE 10 |
|  |  | (b) Indicate number of participants with missing data for each variable of interest PAGE 5, second paragraph |
|  |  | (c) Summarise follow-up time (e.g., average and total amount) PAGE 10, second paragraph |
| Outcome data | 15* | Report numbers of outcome events or summary measures over time PAGE 10, second paragraph |
| Main results | 16 | (*a*) Give unadjusted estimates and, if applicable, confounder-adjusted estimates and their precision (e.g., 95% confidence interval). Make clear which confounders were adjusted for and why they were included PAGES 10, second paragraph, and TABLE 3 |
|  |  | (*b*) Report category boundaries when continuous variables were categorized NOT APPLICABLE |
|  |  | (*c*) If relevant, consider translating estimates of relative risk into absolute risk for a meaningful time period TABLE 3 |
| Other analyses | 17 | Report other analyses done—eg analyses of subgroups and interactions, and sensitivity analyses PAGES 11, second and third paragraphs, PAGE 12, first and second paragraph, FIGURE 1 |
| Discussion | | |
| Key results | 18 | Summarise key results with reference to study objectives PAGES 12, third paragraph |
| Limitations | 19 | Discuss limitations of the study, taking into account sources of potential bias or imprecision. Discuss both direction and magnitude of any potential bias PAGES 15, third paragraph, PAGE 16, first paragraph |
| Interpretation | 20 | Give a cautious overall interpretation of results considering objectives, limitations, multiplicity of analyses, results from similar studies, and other relevant evidence PAGES 12, fourth paragraph, PAGES 13-16 |
| Generalisability | 21 | Discuss the generalisability (external validity) of the study results PAGE 15, third paragraph, PAGE 16 |
| Other information | | |
| Funding | 22 | Give the source of funding and the role of the funders for the present study and, if applicable, for the original study on which the present article is based PAGE 17 |

*Give information separately for exposed and unexposed groups.

**Note:** An Explanation and Elaboration article discusses each checklist item and gives methodological background and published examples of transparent reporting. The STROBE checklist is best used in conjunction with this article (freely available on the Web sites of PLoS Medicine at http://www.plosmedicine.org/, Annals of Internal Medicine at http://www.annals.org/, and Epidemiology at http://www.epidem.com/). Information on the STROBE Initiative is available at http://www.strobe-statement.org.
